# National culture as a correlate of research output and impact

**DOI:** 10.12688/f1000research.18283.3

**Published:** 2020-02-07

**Authors:** Juneman Abraham

**Affiliations:** 1 Psychology Department, Faculty of Humanities, Bina Nusantara University, Jakarta, 11480, Indonesia

**Keywords:** research impact, research productivity, national culture, individualism, indulgence, power distance, citations per document, self citations

## Abstract

National culture has been overlooked in discussions related to research output and impact owing to individual, socio-political structure, and economic factors. This study shows the relationships between the dimensions of cultural value orientation of the nation and research output & impact. More than 60 countries were included, and Spearman correlation analysis was employed. The variables were taken from Geert Hofstede and Scimago Journal & Country Rank worksheets. This study found that (1) Power distance - the positive inclination of the culture toward power disparities among people - is negatively correlated with research impact; (2) Individualism - the level of independence a society keeps up among its individuals -  are positively correlated with research output and research impact; (3) Indulgence - the degree to which society members do not attempt to control their urges - is positively correlated with research impact; and (4) after controlling the Log GDP per capita, uncertainty avoidance - the  manner in which that a society seeks to manage the actuality that the future can never be controlled - is negatively correlated with research impact.

## Introduction

Makri (2018) recently released a report on the increasing number of publications in various countries. She stated that it’s unclear what has triggered and driven the strong gains in Egypt and Pakistan. Throughout the report, various variables believed to be responsible for the increasing number of publications, such as indexation duration, funding, global engagement, international collaboration, and political policies on science and higher education, are explained.

Several predictors of research output and impact had been identified, i.e. author characteristics, co-authorship networks, citation history, journal impact factors, tweets ( Xiaomei *et al.*, 2017), cohort effects (in terms of scientific discipline), age, career stages, gender, the country of origin of the PhD holders, and reward structure of the research enactment ( Claudia & Francisco, 2007). They are mostly at the individual and institutional level. At the country level, the predictors are the number of universities, GDP per capita, control of corruption, civil liberties ( Mueller *et al.*, 2016), country’s wealth and population size, country’s value of research tradition, tenure and promotion criterion, experimental costs, IRB (Institutional Review Boards) review flexibility, language barrier, and the training of new young researchers ( Demaria, 2009).

However, national cultural orientation (in this paper, the term is used interchangeably with: national culture, national cultural value, national culture dimension) is yet to be analyzed, with the present study assuming that individual, institutional, and structural factors are also influenced by the cultural values of a nation. Hofstede Insights (2019) defined culture as the collective mental programming of the human mind which distinguishes one group of people from another, consisting of six dimensions, i.e. (1) power distance (PDI) – acceptance on the unequal power distribution in a society; (2) uncertainty avoidance (UAI) – intolerance of ambiguity and uncustomary thoughts and practices; (3) individualism (IDV) – projection of individuals’ “I” in a society rather than “we” (collectivism); (4) masculinity (MAS) – the toughness and competitiveness rather than the tenderness and cooperativeness (femininity) orientation; (5) long term orientation (LTOWVS) – the society’s preference of time-honored rather than pragmatic approaches (short term normative orientation); and (6) indulgence (IVR) – the society facilitation towards a fun and enjoyable life rather than restraint (suppression of needs gratification by strict social norms).

National culture is relatively stable ( Maseland & van Hoorn, 2017) and is widely used to explain various performances at the country level, such as learning and academic performance ( Signorini *et al.*, 2009). The present study hypothesized that there are correlations between the national culture dimensions and research performance indicators, i.e. research output and impact. The research performance is assumed to be mediated by research culture, and the culture experiences stimulations and challenges from the national culture.

## Methods

All following data were retrieved on August 18, 2019, and compiled into a worksheet (see Underlying data ( Abraham, 2019) as the material of this present analysis. Countries’ region, total documents/DOC, citable documents/CITA, citations/CIT, self-citations/SELF, H-index/HINDEX, and citations per document/CPD (1996–18 August 2019) were obtained from the Scimago Journal & Country Rank/SCIMAGOJR ( https://www.scimagojr.com/countryrank.php?out=xls), while national cultural orientations ( *PDI*=power distance, *IDV*=individualism, *MAS*=masculinity, *UAI*=uncertainty avoidance, *LTOWVS*=long term orientation, *IVR*=indulgence) were acquired from Geert Hofstede web site ( https://geerthofstede.com/wp-content/uploads/2016/08/6-dimensions-for-website-2015-08-16.xls). Countries’ GDP per Capita (1993–2018) were taken from the World Bank Open Data ( http://api.worldbank.org/v2/en/indicator/NY.GDP.PCAP.PP.CD?downloadformat=excel), being calculated as natural logarithm ( *ln*) of the average measures.

Principal component analysis (PCA) and Independent-samples Kruskal-Wallis H Test were done using *IBM SPSS Statistics version 25 for Windows* to get two major components from dimensions reduction of DOC, CITA, CIT, SELF, HINDEX, and CPD, as well as comparison between countries’ regions in terms of the reduced dimensions. Correlation analysis was conducted using *JASP version 0.10.2 for Windows*, and Partial correlation analysis was conducted using *IBM SPSS Statistics*.

## Results and Discussion

The purpose of this study is to show whether there are correlations between national cultural values and research output and impact. Because correlation is not causation, the following analysis and interpretation do not attempt to state definitively that there is a causal effect from one variable to another. Even though in this discussion cultural value orientation is often used as an explanation of research output and impact, this is more due to the chronological flow that culture comes and envelops, engulfs a country first than the SCIMAGOJR measures ( Table 1). The argument is in line with the proposition of Sen (2004) that culture is a constituent of development and economic behavior, as expressed as follows:

**Table 1.** Descriptive statistics of SCIMAGOJR indicators (1996–18 August 2019).

	DOC	CITA	CIT	SELF	CPD	HINDEX
Valid	239	239	239	239	239	239
Missing	0	0	0	0	0	0
Mean	226870.448	208895.238	4.041e +6	1.209e +6	14.289	191.904
Std. Error of Mean	62573.389	56718.779	1.372e +6	584905.378	0.451	17.893
Std. Deviation	967361.125	876851.047	2.122e +7	9.042e +6	6.967	276.624
Variance	9.358e +11	7.689e +11	4.501e +14	8.177e +13	48.541	76521.054
Shapiro-Wilk	0.231	0.237	0.172	0.106	0.905	0.637
*p* of Shapiro- Wilk	< .001	< .001	< .001	< .001	< .001	< .001
Minimum	2.000	1.000	9.000	0.000	2.000	1.000
Maximum	1.207e +7	1.070e +7	2.977e +8	1.344e +8	52.300	2222.000

DOC = Total documents; CITA = Total citable documents; CIT = Total citations; SELF = Total self-citations; CPD = Citations per document; HINDEX = H-index. The operational definition of DOC, CITA, CIT, SELF, CPD, and HINDEX could be found at https://www.scimagojr.com/help.php

“The furtherance of well-being and freedoms that we seek in development cannot but include the enrichment of human lives through … forms of cultural expression and practice, which we have reason to value …. Cultural influence can make a major difference to work ethics, responsible conduct, spirited motivation, dynamic management, entrepreneurial initiatives, willingness to take risks, and a variety of other aspects of human behavior which can be critical to economic success.” (pp. 39–40).

In other words, culture can influence public policy which regulates human capital; whereas, research output and impact depend on human capital, in addition to the fact that research is a contributor to economic growth and development ( Blanco *et al.*, 2015). However, this study is cautious for not trapping itself in cultural determinism.

**Figure 1.**
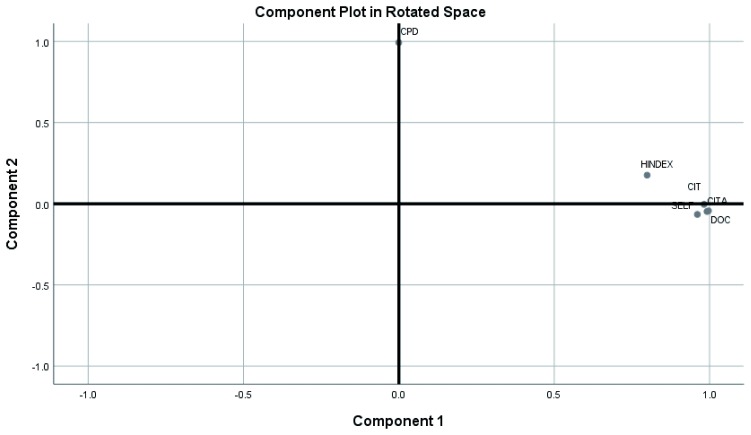
Component plots.

A Principal Components Analysis (PCA) was done resulting in two components extracted with a total variance explained 92.073% ( Table 2), namely:

- **Component 1: “** *Research Output* **”** (a synthesis of DOC, CITA, CIT, SELF, HINDEX). This component comprises of volume-dependent measures (i.e., measures that expand with the quantity of publications of a country).
- **Component 2: “** *Research Impact”* (based on CPD alone). This component comprises of a volume-free measure (i.e., a measure that is autonomous of the quantity of publications of a country). The correlation between Component 1 and Component 2 is weak (< 0.2; see also the plots of the indicators in Figure 1). It might be that CPD is more difficult to manipulate or be an object of the author’s engineering.

**Table 2.** Component loadings of principal component analysis.

	Component 1	Component 2	Correlation between Components	KMO of Sampling Adequacy	Bartlett’s Test of Sphericity
CIT	**0.981**	-0.043	0.159	0.662	*χ* ^2^ (15) = 3759.508, *p* = 0.000
CITA	**0.982**	-0.087			
CPD	0.197	**0.974**			
DOC	**0.987**	-0.083			
HINDEX	**0.833**	0.140			
SELF	**0.947**	-0.104			
Variance Explained	75.498%	16.575%			
Name of component given by the author	Research Output	Research Impact			

Applied rotation method is direct oblimin; KMO ( *Kaiser-Meyer-Olkin* > 0.6) and Bartlett’s ( *p* < 0.05) assumption were fulfilled; DOC = Total documents; CITA = Total citable documents; CIT = Total citations; SELF = Total self-citations; CPD = Citations per document; HINDEX = H-index

Descriptive statistics of SCIMAGOJR measures ( Table 1) showed that the research output (DOC, CITA, CIT, SELF, HINDEX) and impact (CPD) data are not normally distributed ( *p* of Shapiro-Wilk < .05). Therefore, correlation analysis was done with Spearman’s correlation.

In anticipating the inflated type-1 error, the analysis employed significance level of *q* (adjusted *p*) = 0.00714. The four results ( Table 3) are as follows:

***First*, Power Distance (PDI) is negatively correlated with Research Impact.** This could be because PDI negatively correlates with democracy ( Maleki & Hendriks, 2014). The lower level of democracy reduces the opportunity of the academic community to exchange and market (in the broad sense) scientific information, as well as debate openly. Likewise, democracy that does not flourish deters the use of research results in creating public policies. Science is co-opted or used as just a tool to achieve exclusive interests by ideologues, pundits, and political leaders; they ignore the state-of-the-art nature of the research ( Branscomb & Rosenberg, 2012). In addition, PDI might manifest itself in academic writing in the form of rigid, authoritative, defensive, and dogmatic styles ( Koutsantoni, 2005). All the conditions could reduce research impact.

**Table 3.** Correlations results between national cultures dimensions and research output and impact.

		PDI		IDV		MAS	UAI	LTOWVS		IVR		LGDP		Research		Research	
														Performance		Impact	
PDI	ρ	—															
	*p*	—															
	*q*	—															
	95% CI	—															
	0.99% CI	—															
IDV	ρ	-0.612		—													
	*p*	< .001	***	—													
	*q*	< .001	****														
	95% CI	-0.742, -0.438		—													
	0.99% CI	-0.613, -0.611		—													
MAS	ρ	0.039		0.101		—											
	*p*	0.750		0.414		—											
	*q*	1.000		1.000													
	95% CI	-0.201, 0.275		-0.141, 0.331		—											
	0.99% CI	0.038, 0.041		0.099, 0.102		—											
UAI	ρ	0.268		-0.237		-0.206	—										
	*p*	0.027	*	0.051		0.092	—										
	*q*	0.567		1.000		1.000											
	95% CI	0.032, 0.476		-0.450, 0.001		-0.424, 0.034	—										
	0.99% CI	0.267, 0.270		-0.239, -0.236		-0.207, -0.204	—										
LTOWVS	ρ	0.028		0.124		-0.042	-0.030	—									
	*p*	0.829		0.339		0.746	0.816	—									
	*q*	1.000		1.000		1.000	1.000										
	95% CI	-0.223, 0.276		-0.130, 0.362		-0.289, 0.210	-0.278, 0.221	—									
	0.99% CI	0.026, 0.030		0.122, 0.125		-0.044, -0.040	-0.032, -0.028	—									
IVR	ρ	-0.363		0.173		0.138	-0.100	-0.441		—							
	*p*	0.004	**	0.183		0.290	0.443	< .001	****	—							
	*q*	0.100		1.000		1.000	1.000	< .001									
	95% CI	-0.563, -0.122		-0.083, 0.407		-0.118, 0.377	-0.343, 0.156	-0.595, -0.255		—							
	0.99% CI	-0.364, -0.361		0.171, 0.174		0.136, 0.139	-0.102, -0.098	-0.442, -0.440		—							
LGDP	ρ	-0.609		0.663		0.057	-0.222	0.245	*	0.372		—					
	*p*	< .001	***	< .001	***	0.648	0.071	0.021		< .001	***	—					
	*q*	< .001	****	< .001	****	1.000	1.000	0.462		< .001	****						
	95% CI	-0.741, -0.432		0.503, 0.779		-0.186, 0.293	-0.439, 0.019	0.039, 0.431		0.539		—					
	0.99% CI	-0.610, -0.608		0.662, 0.664		0.055, 0.058	-0.224, -0.221	0.244, 0.246		0.181, 0.178		—					
Research	ρ	-0.332		0.528		0.205	-0.231	0.272		0.371, 0.373		0.465		—			
Performance	*p*	0.006	**	< .001	***	0.093	0.058	0.009	**	0.086		< .001	***	—			
	*q*	0.144		< .001	****	1.000	1.000	0.207		1.000		< .001	****				
	95% CI	-0.528, -0.101		0.332, 0.681		-0.035, 0.423	-0.445, 0.007	0.069, 0.454		-0.026, 0.373		0.346, 0.570		—			
	0.99% CI	-0.333, -0.330		0.527, 0.529		0.204, 0.207	-0.233, -0.230	0.271, 0.273		0.180, 0.183		0.465, 0.466		—			
Research	ρ	-0.609		0.497		-0.007	-0.107	-0.054		0.508		0.120		0.288		—	
Impact	*p*	< .001	***	< .001	***	0.956	0.387	0.616		< .001	***	0.098		< .001	***	—	
	*q*	< .001	****	< .001	****	1.000	1.000	1.000		< .001	****	1.000		< .001	****		
	95% CI	-0.740, -0.434		0.294, 0.658		-0.245, 0.232	-0.336, 0.135	-0.258, 0.155		0.338, 0.647		-0.022, 0.258		0.167, 0.400		—	
	0.99% CI	-0.610, -0.608		0.496, 0.499		-0.008, -0.005	-0.108, -0.105	-0.055, -0.052		0.507, 0.509		0.120, 0.121		0.287, 0.288		—	

DOC = Total documents (1996–18 August 2019); CITA = Total citable documents; CIT = Total citations; SELF = Total self-citations; CPD = Citations per document; HINDEX = H-index; PDI = Power distance; IDV = Individualism (vs. Collectivism); IVR = Indulgence (vs. Restraint); LGDP = Natural logarithm of averaged (1993–2018) GDP per capita. *ρ* = Spearman’s rho; * *p* < 0.05; ** *p* < 0.01; *** *p* < 0.001; *q* = adjusted *p*-values ( Gaetano, 2018; Holm, 1979); the significance level is 0.00714; **** *q* < 0.00714; CI = Confidence Interval; 0.99% CI = 0.99286% CI.

***Second*, Individualism (IDV) is positively correlated with Research Output and Research Impact.** The positive correlations could be explained using the findings of Deschacht & Maes (2017). They found that in countries with more individualistic cultures: (1) the scientists prioritize their self-development, (2) the records of scientific work are historically longer (usually Western countries), and (2) self-citations flourish more. This does not necessarily mean that there have been citation abuses, but that self-citation is used to refer to their prior works, thereby, preventing unnecessary repetitions of ideas in newer works ( Deschacht, 2017). Although IDV and collaboration are often contested (e.g. Kemp, 2013), a “collaborative individualism” ( Limerick & Cunnington, 1993) – stressing both working together and self-emancipation – is possible, explaining the positive correlation.

***Third*, Indulgence (IVR) is positively correlated with Research Impact.** This may be because IVR – the warranted one – facilitates academic freedom ( Ohmann, 2011), as stated by Jefferson (2011) regarding psychological gratification, “Difference of opinion is advantageous … [F]ree inquiry must be indulged, and how can we wish others to indulge it while we refuse it ourselves” (p. 26). Conversely, a restraint (as opposed to indulgence) will facilitate the destruction of goal pursuit, e.g. designing and executing impactful studies, through psychological reactance and unwarranted indulgence ( Buzinski & Price, 2015). Sabbatical leave is a representative example of warranted IVR that faculty members could increase research impact through the special time ( Robert Gordon University, 2016). Through the leave, faculty members are temporarily freed from normal academic routines and intensively entering the real world where social decisions and policy makings occur. The various experiences expressed in Harvey Mudd College (2019) showed that in undergoing sabbatical leave, faculty members really enjoyed their social, recreational, and cultural adventures, supporting their research life. All the conditions could increase the research quality and, eventually, research impact. In addition, IVR facilitates open science, because, in the perspective of open science, science is indeed an art ( Fleming, 2019; Kera, 2017; Thornton, as cited in BBC News, 2010). Meanwhile, open science practices (such as research sharing through social media and even cartoons and drawings, data archiving and aggregation, team-science, crowd and shared databases, replicability and repeatability improvement efforts, very big data curation and management, engagement with research stakeholders) could enhance research impact in terms of citations per document (CPD) (e.g. De Filippo & Sanz-Casado, 2018) even in terms of the economy of research ( Adams, 2015). This is because open science increases public esteem in science. IVR may also manifest itself in a “lovely” academic writing style ( Kiriakos & Tienari, 2018). This style is not dry and cold, but rather dialogical, humanistic, more reflexive, and capable of showing authors’ courage and vulnerability. Compelling insights are more easily born from the writings that embody those qualities; as mentioned, “a thin line exists between interesting insights and self-indulgence” ( Nadin & Cassell, 2006, p. 214). Scientific authors who read such works would be attracted to cite them, leading to an increase in the works’ impact. In addition, “strategic indulgence” is possible and known to be a creative process that enables one to balance academic activity (such as writing) with non-academic ones ( Jia *et al.*, 2018) – fostering insight.

***Fourth*, LGDP is positively correlated with Research Output.** This is in line with the finding of Mueller *et al.* (2016), that economic prosperity (per capita GDP) is one of the best predictors of the country’s research output.

Partial correlation by controlling LGDP showed that the directions of correlation between variables are the same as the results of Spearman's correlation above ( Table 3), but there is an additional new result ( Table 4). **Uncertainty Avoidance (UAI) is found negatively correlated with Research Impact**. This is understandable considering that impactful research requires innovation. The characteristics of UAI - which are intolerant of ideas and practices that are ambiguous and not conventional - do not support innovation ( Bauer & Suerdem, 2016). Uncertainty avoidance cultural orientation is difficult to challenge and scrape unfunctional attitudes and values that are already stable. Therefore, it will also be hard to produce breakthroughs in research and publication, reducing the potential for citations per document. One premise advocated by Leiden Manifesto for Research Metrics is “Science and technology indicators are prone to conceptual ambiguity and uncertainty and require strong assumptions that are not universally accepted” ( Hicks *et al.*, 2015, para. 21). Higher UAI national culture would adhere to the invariance assumption that is detrimental to the development of science and publication real impact. Un-openness to the pluralistic approach in the impact measurement will invite citation cartels. Citations per document (CPD) will be seen reductionistically as the destination of scientific works, so that CPD will be easy to become a target of manipulation. In fact, we have been reminded that the production of knowledge and its memories must not forget the relevance of knowledge to diverse publics. What is needed is a “careful and conscientious citation ... [citation as] a form of engagement”, in which “citation as a crude measure of impact” is only the byproduct of the reflexive action ( Mott & Cockayne, 2017, p. 2, 11). It will need lower UAI.

**Table 4.** Partial correlations between national cultures dimensions and research output and impact, controlling Log GDP per capita (LGDP) ( *N* = 60, *df* = 57).

		PDI	IDV	MAS	UAI	LTOWVS	IVR
Research Output	*r*	-0.061	0.303	0.201	-0.176	0.011	0.026
	*p*	0.648	0.019 *	0.127	0.183	0.932	0.847
Research Impact	*r*	-0.495	0.432	-0.086	-0.261	-0.112	0.273
	*p*	0.000 ***	0.001 **	0.518	0.046 *	0.397	0.037 *

### Research output across regions

Descriptive statistics of national culture, research output, and research impact ( Table 5) showed that the data are not normally distributed (most of the *p* of Shapiro-Wilk < .05). Therefore, comparison of the research indicators between regions was analyzed with Kruskal-Wallis H Test.

**Table 5.** Descriptive statistics of national cultural orientations, research output and impact.

	PDI	IDV	MAS	UAI	LTOWVS	IVR	LGDP	Research Output	Research Impact
Valid	68	68	68	68	90	91	190	239	239
Missing	171	171	171	171	149	148	49	0	0
Mean	59.118	43.853	48.603	67.132	46.067	45.374	9.008	1.004e -17	4.766e -16
Std. Error of Mean	2.671	2.930	2.420	2.820	2.560	2.364	0.088	0.065	0.065
Std. Deviation	22.023	24.164	19.956	23.257	24.287	22.555	1.208	1.000	1.000
Variance	485.031	583.918	398.243	540.893	589.838	508.748	1.460	1.000	1.000
Shapiro-Wilk	0.985	0.942	0.980	0.951	0.968	0.978	0.979	0.264	0.915
*p* of Shapiro-Wilk	0.587	0.003	0.334	0.010	0.024	0.120	0.006	< .001	< .001
Minimum	11.000	6.000	5.000	8.000	0.000	0.000	6.442	-0.303	-1.800
Maximum	104.000	91.000	110.000	104.000	100.000	100.000	11.614	12.810	5.201

PDI = Power distance; IDV = Individualism (vs. Collectivism); MAS = Masculinity (vs. Femininity); UAI = Uncertainty avoidance; LTOWVS = Long term (vs. Short term) Normative Orientation; IVR = Indulgence (vs. Restraint); LGDP = Natural logarithm of averaged (1993–2018) GDP per capita; Research Output = Z-scores of Component 1 from Principal Component Analysis/PCA extraction (based on DOC, CITA, CIT, SELF, HINDEX); Research Impact = Z-scores of Component 2 from PCA extraction (based on CPD); PCA = Principal Component Analysis

Kruskal-Wallis H Test showed results as follows:

Mean comparisons result in terms of Research Output (details in Table 6):

- Eastern Europe > Latin America
- Eastern Europe > Pacific Region
- Eastern Europe > Africa
- Middle East > Latin America
- Middle East > Pacific Region
- Middle East > Africa
- Asiatic Region > Pacific Region

Mean comparisons result in terms of Research Impact (details in Table 6):

- Latin America > Middle East
- Latin America > Asiatic Region
- Latin America > Eastern Europe

**Table 6.** Comparison of research output and impact between regions.

Variable	Test statistic	Visualization of mean rank	Pairwise comparison
Research output (Component 1)	*χ* ^2^ (6, *N* = 204) = 41.952, *p* = 0.000	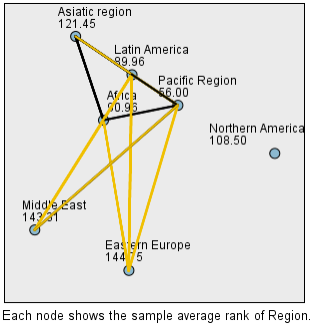	There are significant differences (marked with yellow lines in the visualization) between: • Pacific Region & Middle East ( *p* = 0.000) • Pacific Region & Eastern Europe ( *p* = 0.000) • Latin America & Middle East ( *p* = 0.038) • Latin America & Eastern Europe ( *p* = 0.005) • Africa & Middle East ( *p* = 0.036) • Africa & Eastern Europe ( *p* = 0.004) • Pacific Region & Asiatic Region ( *p* = 0.01)
Research Impact (Component 2)	*χ* ^2^ (6, *N* = 204) = 29.363, *p* = 0.000	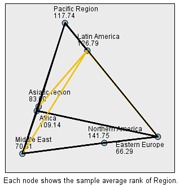	There are significant differences (marked with yellow lines in the visualization) between: • Middle East & Latin America ( *p* = 0.020) • Asiatic Region & Latin America ( *p* = 0.023) • Eastern Europe & Latin America ( *p* = 0.001)

*p*-values have been adjusted by the Bonferroni correction for multiple tests; the significance level is 0.05

**Figure 2.**
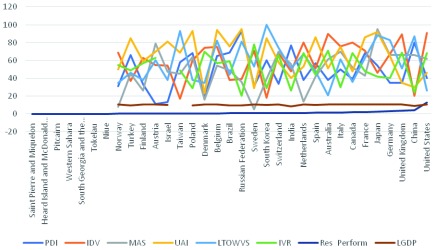
Plots of national cultures, research output, and Log GDP per capita. PDI = Power distance; IDV = Individualism; MAS = Masculinity; UAI = Uncertainty avoidance; LTOWVS = Long term orientation; IVR = Indulgence; Res_Perform = Research Output; LGDP = Log GDP per capita.

**Figure 3.**
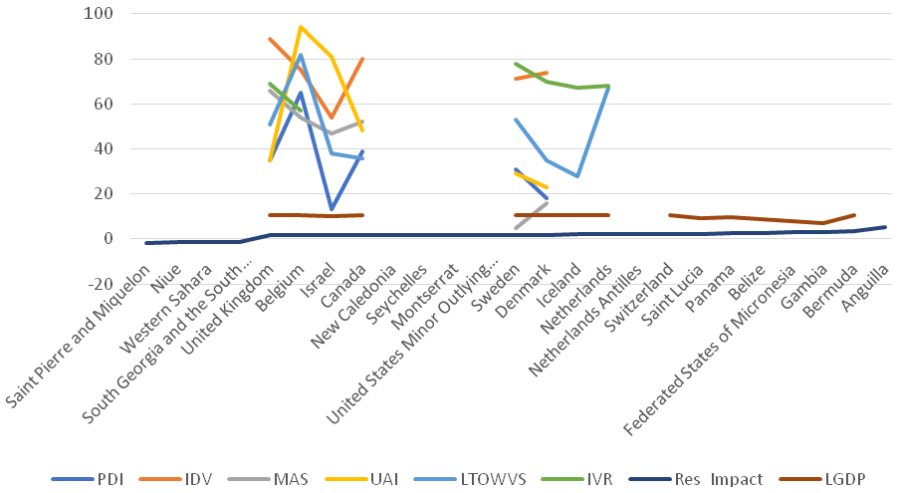
Plots of national cultures, research impact, and Log GDP per capita. PDI = Power distance; IDV = Individualism; MAS = Masculinity; UAI = Uncertainty avoidance; LTOWVS = Long term orientation; IVR = Indulgence; Res_Perform = Research Output; LGDP = Log GDP per capita.

Based on the comparisons between regions over the past 23 years (1996–2019) (1) both Eastern Europe and Middle East have better research output than Latin America, Pacific Region, and Africa; (2) Asiatic Region has better research output than Pacific Region. However, from the aspect of research impact, Latin America outperforms the Middle East, Asiatic Region, and Eastern Europe. Those findings show that research output and research impact are not always directly proportional, they can even be inversely correlated (see also Figure 2, Figure 3).

Eastern Europe’s superiority in terms of research output may be due to the rise of democracy, the emergence of the need for research excellence standards, the promotion of international research collaboration, and cooperation with international bodies (such as the World Bank) that enable these countries to enjoy large research grants ( Henderson *et al.*, 2012; Švab, 2004).

Henderson *et al.* (2012) further stated a fact about research culture in Eastern Europe, as follows:

“Though not a uniform phenomenon across all disciplines or countries, some participants noted that in CEE (Central and Eastern Europe) research tends to be more dependent on political power. This can relate both to the partisan provision of financial resources and to researchers’ ambitions to convince political actors.”

It appears that political activities are melting pots of the interests of academics, politicians, and research funders, which provide work opportunities that has implication in improving research output in the region’s countries. Those interests are given “energy” by the belief of the people that “Our people are not perfect, but our culture is superior to others.” ( Kim, 2018, para. 6).

Makri’s finding (2018) regarding the progressive research achievement of Egypt confirmed the finding that the Middle East has been able to surpass Latin America, the Pacific Region, and Africa in terms of research output. Different from Eastern Europe, the output in Middle East has drivers centripetalized on the publishing business. Although some of the Middle East countries are plagued with protracted conflict ( Gul *et al.*, 2015), Habibzadeh (2019) noted that there is a “meeting point” between the career interests of faculty members in universities and the business interests of publishing in the countries. This is exacerbated by the relaxation of the promotion standard of faculty members, so that a surge in publication occurs in Scopus indexed journals—that grow rapidly quantitatively in those countries, but of which many have transformed into predatory ones. Habibzadeh (2019, p. 4) conveyed more about the phenomenon:

“Recently, some indexing systems, like Scopus, have also pursued the same strategy and delisted some of the low-quality journals published in the Middle East and Iran. Although some of the editors and publishers of the delisted journals have attributed these events to political issues, to be honest, I, for one, believe that in most instances, they, themselves, should bear the brunt of the situations they have for their poor work quality.” (p. 4)

Noteworthy is the fact mentioned by Plackett (2015), that:

“The predatory journal industry exists on a spectrum—at one end, some such journals maintain they are conducting valid peer review. At the other end of the spectrum, predatory journals sometimes blackmail academics who eventually realize they’ve published in a journal with a negative reputation.” (para. 21)

That is, the issue of predatory journals in the Middle East is not an easy problem to evaluate. This argument is reinforced by Jones’ (2015) argument, that the flourish of predatory journals is not the real problem. The fundamental problem, according to Jones, is information inequality; in which case, the prosocial role of librarians and publishers to keep potential writers away from illegitimate journals may still be difficult to expect. It is not surprising that, based on the results of this present study, even though research output of Middle East outperforms Latin America, in terms of research impact, the opposite occurs, i.e. Latin America outperforms the Middle East, also the Asiatic Region, and Eastern Europe.

According to SCIMAGOJR data ( https://www.scimagojr.com/countryrank.php?region=Latin%20America&order=cd&ord=desc), retrieved on September 2019, the six countries with the highest combination of documents and citations are Panama, Puerto Rico, Uruguay, Costa Rica, Argentina, and Chile. Related to the literature in these countries, Ward (2016) stated its virtue, “Only with slow, careful, detailed analysis, concern, and empathy even can be liberated from the old ways of seeing” (p. xxiii). These “human qualities” of Latin America’s publications may attract citations repeatedly. This explanation, nevertheless, is still speculative and requires testing in subsequent empirical studies.

Plots of national cultures, research output, and Log GDP per capita ( Figure 2; *missing scores do not bring up the line*) showed that, based on *low vs. high* research output criteria (< -0.30 *σ* vs. > 0.30 *σ*), it is found that, among 33 countries (7 *low* vs. 26 *high*), (1) United States, (2) China, (3) United Kingdom, (4) Germany, and (5) Japan are countries with the highest research output. Descriptively, in each of these countries, the national cultural orientations that play roles the most and the least are, respectively: (1) Individualism, long term orientation; (2) Long term orientation, individualism; (3) Individualism, uncertainty avoidance; (4) Long term orientation, power distance; (5) Masculinity, indulgence. For countries with the lowest research output, there is no data available on their national cultural orientation.

### Research impact across regions

Latin America’s superiority in terms of research impact cannot be separated from the orientation of studies that aspires to decolonize the research itself ( International Institute of Social Studies, 2019), even beginning from the decolonization of consciousness ( Garza, 2010). Decolonization of research in the context of Latin America has the meaning of restoring the authentic identity of society, from an oppressed condition—by “capitalism, hegemony, racism, classism, sexism, etc.” (Garza, p. 110)—to an emancipated situation. There is hope for reconnection of the daily lives of people and their families, communities, and even living creatures, from those that have been being alienated by the oppression. The assumption is, “You actually cannot have meaningful, impactful research unless you engage communities” ( Janes, 2017, p. 114). Studies conducted in Latin America are very directed towards liberating the fate of the society, especially from marginalized conditions in various fields of life, such as in health, agricultural, environmental, social, and other domains.

Meanwhile, the issues of (de-)colonization are studied very seriously by countries that experience a similar fate and become huge energy for doing high impact research. This is because many problems “have been attributed to the impact of ongoing colonization” (Waldram, as cited in Marsh *et al*., 2015, p. 2). The activities of the academic community of Latin America are increasingly supported by the AmeliCA project, namely The Latin American Initiative which focuses on developing scientific communication systems that are non-commercial, academic-led, and cooperative ( Aguado-López & Becerril-Garcia, 2019), so that could improve citations per document of scientific works in Latin America.

Plots of national cultures, research impact, and Log GDP per capita ( Figure 3 ; *missing scores do not bring up the line* showed that, based on *low vs. high* research impact criteria (< -1.50 *σ* vs. > 1.50 *σ*), it is found that, among 25 countries (4 *low* vs. 21 *high*), (1) Anguilla, (2) Bermuda, (3) Gambia, (4) Federated States of Micronesia, and (5) Belize are the countries with the highest research impact. Unfortunately, data are not yet available about the orientation of their cultural values. The complete data (six cultural orientations) available are from (1) Belgium, and (2) United Kingdom. Descriptively, in each of these countries, the cultural orientations that play roles the most and the least are, respectively: (1) Uncertainty avoidance, power distance; (2) Individualism, uncertainty avoidance (as well as power distance). For countries with the lowest research impact, there is no data available on national cultural orientation.

### The limitation of SCIMAGOJR data

There are three things that need to be aware of when reading the results, namely: *First*, the SCIMAGOJR data ( Table 1) includes both journal articles, conference proceedings papers, and does not exclude other types of documents (i.e. short survey, review) ( Guerrero-Bote & Moya-Anegón, 2012). A number of countries or institutions exclude non-journal articles from evaluating their performance (e.g. Suryani *et al*., 2013), so the applicability of the results of this study to these countries might be limited. In this present study, data from SCIMAGOJR is used because, among others, it can be downloaded for free. This limitation may affect the accuracy of research output and impact measurements.

*Second*, in a number of dimensions of research output and impact measurement, Scopus, which supplies the data of SCIMAGOJR, has a number of limitations; for example (1) Scopus has poor coverage of articles, conference papers, and book chapters compared to Crossref, Dimensions, Google Scholar, and Microsoft Academic; (2) Scopus is somewhat late in indexing in-press articles compared to all four; (3) Socially, Scopus does not support open citation ( Harzing, 2019). However, the limitation of Scopus is offset by its advantages, namely Scopus is still an extensive source of quality citation data ( van Eck *et al.*, 2018).

*Third*, SCIMAGOJR, at least in its open access form, does not provide time series data. SCIMAGOJR data is cumulative data at a particular point in time, not annual data. Thus, the results of the correlation of various variables with SCIMAGOJR indicators might be most likely to suffer from long-term influences of background trends. However, the author has made a number of attempts to minimize the possibility of correlational bias. First, the author has found theoretical support confirming that national cultural orientation does not fluctuate much between years, e.g. “ Hofstede *et al.* (2010) compare nations to organisms, citizens to cells, and cultures to DNA .... And cultures, like organisms, can stay consistent for long periods, evolve gradually over time, or adapt to sudden changes” ( Whalen, 2016, p. 4). Second, the LGDP variable was controlled (with partial correlation analysis) because it was realized that the correlation between national cultural orientation and research output and impact might be affected by the country’s economic situation.

## Conclusion

National culture dimensions, especially power distance, individualism, indulgence, and uncertainty avoidance are pivotal variables that are to be considered in justifying research impact. In addition, the only variable that correlates with research output is individualism.

Owing to the fact that the national culture is relatively enduring, countries need to measure their elasticity of hopes and action plans in an effort to boost research output and impact, by integrating the national culture in the estimate. National culture can be integrated as a moderating variable in the predictive relationship between GDP per capita and research output and impact. Diversification of this study – based on the document and authors’ collaboration types, the indexing databases, the disciplines, as well as the history and development of the research in a country – is a future opportunity for further study.

## Data availability

### Source data

Geert Hofstede: Dimension data matrix. https://geerthofstede.com/research-and-vsm/dimension-data-matrix/ ( Hofstede *et al.*, 2010)

Scimago Journal & Country Rank: Download data. https://www.scimagojr.com/countryrank.php?out=xls ( Scimago Lab, 2019)

GDP per capita, PPP (Current International $). http://api.worldbank.org/v2/en/indicator/NY.GDP.PCAP.PP.CD?downloadformat=excel ( World Bank Open Data, 2018)

All source data was accessed and retrieved on the 18/8/2019

### Underlying data

Figshare: National culture, research performance indicators, and log GDP Per capita. https://doi.org/10.6084/m9.figshare.7723211 ( Abraham, 2019)

Data are available under the terms of the Creative Commons Zero “No rights reserved” data waiver (CC0 1.0 Public domain dedication).
