# Supplementary material for: National culture as a correlate of research output and impact
Source: F1000Res. 2020 Feb 7;8:237. Originally published 2019 Feb 28. [Version 3] doi: 10.12688/f1000research.18283.3 (PMC7029754; doi:10.12688/f1000research.18283.3)
Supplement: Peer review history

# Peer review: Reviewer response for version 3

**DOI:** 10.5256/f1000research.24613.r59689

**Published:** 2020-02-17

**Authors:** Ludo Waltman

**Affiliations:** 1 Centre for Science and Technology Studies (CWTS), Leiden University, Leiden, The Netherlands

I am satisfied with version 3 of this manuscript. I don't require any further improvements to be made by the author. The author has done a nice job!

I confirm that I have read this submission and believe that I have an appropriate level of expertise to confirm that it is of an acceptable scientific standard.

# Peer review: Reviewer response for version 3

**DOI:** 10.5256/f1000research.24613.r59688

**Published:** 2020-02-14

**Authors:** Jonathan P. Tennant

**Affiliations:** 1 IGDORE, Berlin, Germany

I think at this stage, asking to do any more work would be asking too much of an already impressive manuscript.

 While I am still a little cautious about the results and the correlations due to the treatment of time series data, I don't think that at the present this should stop this MS from being indexed and more widely used, discussed, and built upon.

 Thank you to the author for being so considerate of the recommendations from myself and the other reviewer, and thank you for such a good piece of work! Banyak selamat!

I confirm that I have read this submission and believe that I have an appropriate level of expertise to confirm that it is of an acceptable scientific standard.

# Peer review: Reviewer response for version 2

**DOI:** 10.5256/f1000research.22695.r54254

**Published:** 2019-11-25

**Authors:** Jonathan P. Tennant

**Affiliations:** 1 IGDORE, Berlin, Germany

From what I can see, the author has made a number of important changes to this MS, which feels much stronger in its present version. This includes:

- Almost completely rewriting the abstract
- Far more additional information on the source data and methods have been added
- Tables summarising the results have been added now, largely in place of text blocks describing the statistical results
- Additional principal component analyses have been performed
- The discussion has been almost entirely rewritten based on this, including a section on limitations of the data and analyses

I note here that Reviewer 2 has also provided some additional comments in their second review. I agree with both of these points, that the labelling of the PCA plots should be checked and made more appropriate, and that the results section would still benefit from a combined text and table presentation. I think this would entail just re-adding some of the deleted text from the previous version. I think that keep the results and discussion sections distinct though would be beneficial, as there is a lot to digest here. The present version is a bit weird with all of the results condensed into one table, referring to further results which are only then first present in the discussion.

 Additional comments

- Looking at the data again, I am still a bit worried that this involves time series data, but they are not being treated as such. Thus, the results are most likely suffering from autocorrelation or long-term impacts of background trends, and require further statistical analysis to be sure of this. I am really only familiar with time series analysis for palaeontological data though, see for example here: https://www.nature.com/articles/ncomms12737#Sec11 ^1^ – I am a bit suspicious that because of this problem, many of the correlation coefficients reported might be artificially higher than what is realistic. I think that this needs to be very carefully considered here. My apologies for not indicating this in the first report, but I could not see the data to check this.
- The limitations section might work better after the rest of the discussion
- My expertise on the intersection between politics and research is quite limited, and I will refrain from commenting on those elements of the discussion. Although they are, at least to me, very interesting!
- As much of the discussion again is based on the results, which I suspect might change given my recommendations for the methods above, I will refrain from commenting on them too much at the present. From what I can gauge though, they seem to be well thought out, contain relevant literature, and do not oversell the results too much (in their present state).

My apologies for asking for potentially more analytical work to be done at this stage. I feel that it is necessary to look at the data through the lens of time though to better understand some of the results being obtained here. Keep up the great work for now!

I confirm that I have read this submission and believe that I have an appropriate level of expertise to confirm that it is of an acceptable scientific standard, however I have significant reservations, as outlined above.

## References

[1] : Sea level regulated tetrapod diversity dynamics through the Jurassic/Cretaceous interval. *Nature Communications*.2016;7(1) : 10.1038/ncomms12737 10.1038/ncomms12737 PMC502580727587285

# Peer review: Reviewer response for version 2

**DOI:** 10.5256/f1000research.22695.r54253

**Published:** 2019-10-14

**Authors:** Ludo Waltman

**Affiliations:** 1 Centre for Science and Technology Studies (CWTS), Leiden University, Leiden, The Netherlands

The author has made various improvements. However, the revised paper suffers from two problems that I would like to request the author to address. The first problem is a major one, while the second one is more minor.

 First, the results section consists of just one sentence and doesn’t properly present and explain the results. Instead, a summary of the results is presented in Table 1 and this table then provides references to other tables and figures. Based on Table 1, I find it difficult to get a good understanding of the results. Without such an understanding, I also struggle to understand the discussion section. My recommendation is to remove Table 1 and to provide a proper presentation of the results in the results section. Since I didn’t manage to properly understand the results, I refrain from commenting on the discussion of the results in the discussion section. The author could also consider combining the presentation and the discussion of the results in a single section called ‘Results and Discussion’.

 Second, in the principal component analysis, I don’t think the first component should be labeled ‘research performance’. The term ‘research performance’ is very general and could mean many different things. Therefore, ‘research performance’ is not a very helpful label for the first component. The essential difference between the two components is that the first one consists of size-dependent variables (i.e., variables that increase with the number of publications of a country) while the second component consists of a size-independent variable (i.e., a variable that is independent of the number of publications of a country). The first component could be labeled ‘research output’ (since all variables depend on the size of the research output of a country), while the second one could be labeled ‘research impact’.

I confirm that I have read this submission and believe that I have an appropriate level of expertise to confirm that it is of an acceptable scientific standard, however I have significant reservations, as outlined above.

# Peer review: Reviewer response for version 1

**DOI:** 10.5256/f1000research.20000.r46347

**Published:** 2019-04-15

**Authors:** Ludo Waltman

**Affiliations:** 1 Centre for Science and Technology Studies (CWTS), Leiden University, Leiden, The Netherlands

This is a short paper that presents a brief but easy-to-understand analysis of the correlation between the cultural orientation of countries and countries’ scientific performance in terms of bibliometric statistics.

 It would be helpful if the author could offer more extensive information on the way in which the variables used in the analysis have been obtained. The definitions of the bibliometric variables need to be carefully explained. Likewise, it should be explained how the variables for national cultural orientations have been obtained.

 The statistical analysis is carried out using Pearson correlation analysis. Before presenting the correlations, the author should present descriptive statistics for the variables included in the analysis. In particular, I would like to know whether some of the variables may be highly skewed. If this is the case, the use of Pearson correlation analysis could easily lead to misleading results. The use of for instance the Spearman correlation may then be more appropriate.

 The author relies strongly on statistical significance testing. My recommendation is to leave out all significance tests and instead to present confidence intervals for the correlation coefficients. Significance testing leads to problematic dichotomous thinking, as has for instance been pointed out in a recent contribution in Nature (Amrhein et al. ^1^). Following the so-called estimation statistics approach, reporting confidence intervals is preferable over reporting significance tests ( https://en.wikipedia.org/wiki/Estimation_statistics). I am aware that another reviewer (Tennant) recommends performing even more significance tests. I disagree with this recommendation. I don’t consider this to be good statistical practice.

 It would be nice if the author could deepen the analysis a bit more. This can for instance be done by showing scatter plots for the most interesting relationships between variables. In these scatter plots, the names of countries could be shown, especially for those countries that seem to display interesting behavior (e.g., outliers). This would lead to a more in-depth analysis that probably offers richer insights.

 The paper uses lots of abbreviations. This makes the paper more difficult to read. My recommendation is to reduce the number of abbreviations that are used. It may also be helpful to include a table listing all abbreviations and the corresponding full terms.

 The author interprets the analysis in terms of correlation instead of causation. This is very good. There is a risk, however, that some readers may give causal interpretations to the findings of the author. My suggestion is to add a sentence at the end of the paper emphasizing that causal interpretations are not warranted.

I confirm that I have read this submission and believe that I have an appropriate level of expertise to confirm that it is of an acceptable scientific standard, however I have significant reservations, as outlined above.

## References

[1] : Scientists rise up against statistical significance. *Nature*.2019;567(7748) : 10.1038/d41586-019-00857-9 305-307 10.1038/d41586-019-00857-9 30894741

# Peer review: Reviewer response for version 1

**DOI:** 10.5256/f1000research.20000.r46349

**Published:** 2019-03-28

**Authors:** Jonathan P. Tennant

**Affiliations:** 1 IGDORE, Berlin, Germany

The author presents an interesting piece of insight into research impact through a cultural lens, which is quite distinct from a lot of more recent studies which tend to focus on ‘academic impact’ metrics. As a short note, I found it useful in exposing a different dimension to the ongoing debates around research impact. Given my area of “expertise”, I feel that someone who understands social research impact more could provide a great deal of additional insight here during the review process.

**Data**

- The data are included in Figshare, as well as summarized in integrated tables.
- I note that there are a lot of missing data included though, is this just a case of availability?
- Also, I note that the Scimago database is based on Scopus data, which tends to be biased in a number of dimensions. Is it possible to make a note of this?

**Abstract**

- The abstract jumps right into results around Individualism and Power distance and indulgence, without describing what these are (even briefly). This makes it difficult to understand for readers who are perhaps unfamiliar with these concepts. Perhaps a brief explanation of these could be added instead of describing the methods and the data sources, which aren’t really needed?

**Introduction**

- Just to pull out the ‘correlation does not imply causation’ card here; just because there is a correlation between number of publications and other external factors, does not imply a causal relationship necessarily.
- There are a couple of typos (e.g.‘twits’) that might just need a quick copy edit to fix.
- I think the Introduction does a nice job of describing the previous research, and situates the present report well within that.
- Not sure if the comment about China at the end of the Introduction adds too much here.

**Materials and methods**

- So the methods are pretty simple, which is nice. But also, I think perhaps a bit too simple here given that you’re performing a lot of bivariate analyses, and a couple of extra steps are recommended.
  - First, you want to perform an assessment of normality for data series prior to any correlation analyses, using the Shapiro–Wilk test (e.g.,shapiro.test function in R). From the output, if the p-values are greater than the pre-defined alpha level (traditionally, 0.05) this implies that the distribution of the data are not significantly different from a normal distribution, and therefore you can assume normality and use Pearson’s test (Pearson’s product moment correlation coefficient [r]). If p > 0.05, you should instead perform a non-parametric Spearman’s rank correlation (ρ).
  - Secondly, once you’ve done this, for each test, report both the raw and adjusted p-values. The latter can be calculated using the p.adjust() function, and using the ‘BH’ model (Benjamini & Hochberg, 1995 ^1^). This method accounts for the false-discovery test when performing multiple hypothesis tests with the same data set, which can inflate type-1 error (i.e. in order to avoid falsely rejecting a true null hypothesis; a false positive). What this will probably do is reduce the ‘significance’ of some of your results too (which is why it’s best to report both the raw and adjusted values).
- In addition to this, it seems like you have multivariate data, so multivariate analyses might be more informative here. I would strongly recommend performing a Principal Components Analysis on your data (perhaps just only with the variables with more complete data), and inspecting that as a compliment to the bivariate ones. This is fairly easy to do and display using in built functions in R.

**Results**

- I expect that the results will change a bit given my above recommendations to the methods, so won’t comment too much on them at this stage. The nice thing about PCA though is that it produces good summary plots, which might be useful here.
- In the text, can the country abbreviations be given to make reading a bit easier?
- M, SD, and N I think need explaining here too. Lots of acronyms can get a bit confusing!

**Discussion and conclusions**

- As above, I don’t want to comment too much on the Discussion and Conclusions at the present, as I think the above recommended methods will change some of the interpretations. However, at the present there seems to be a logical progression between reported results and conclusions.

Congratulations to the author on a great and interesting piece of work. I would be happy to see a revised version of this too if needed.

I confirm that I have read this submission and believe that I have an appropriate level of expertise to confirm that it is of an acceptable scientific standard, however I have significant reservations, as outlined above.

## References

[1] : Controlling the False Discovery Rate: A Practical and Powerful Approach to Multiple Testing. *Journal of the Royal Statistical Society. Series B (Methodological)*.1995;57: Reference source
